# Re: Repair Index in Examination of Nuclear Changes in the Buccal Mucosa of Smokers: A Useful Method for Screening of Oral Cancer

**DOI:** 10.31557/APJCP.2019.20.11.3189

**Published:** 2019

**Authors:** Armen Nersesyan

**Affiliations:** *Institute of Cancer Research, Medical University* of Vienna, Austria.

**Dear Editor**

A publication by Fahradi et al., (2017) describes the results of the study concerning micronucleus (MN) assay in buccal cells of smokers, a problem which was investigated by many scientists recently (Nersesyan et al., 2006; Blaszczyk and Mielzynska-Svach, 2010; Nersesyan et al., 2011; Chandirasekar et al., 2014; Singaravelu and Sellappa, 2015; de Geus et al., 2018). But this problem is not solved, and MN induction in smokers is still questionable since both positive and negative results were reported in these studies (Nersesyan et al., 2011; Chandirasekar et al., 2014; de Geus et al., 2018; de Geus et al., 2019; Nersesyan, 2019). The authors studied along with MN also nuclear anomalies and presented the results of calculation of so-called “*repair index*”.

The main shortcoming of this publication (very serious one!) is that the authors scored only 500 cells per subject which were stained with Papanicolau stain (which is not DNA specific). I wonder why vast majority of scientists from some Asian counties (namely India) and Latin America (namely Brazil) ignore the validated and standardized buccal MN assay protocol (Thomas et al., 2009). In this protocol is clearly stated that 2,000 buccal cells stained with DNA-specific stain should be scored to get reliable results (Thomas et al., 2009). It is suggested in several publications that for the monitoring of genotoxic effects of carcinogens in exfoliated humans cells 3,000 - 10,000 cells per subject should be evaluated due to the lower baseline MN frequency (Belien et al., 1995; Albertini et al., 2000). Recently, Ceppi et al., (2010) also calculated the minimum number of cells which should be evaluated to obtain reliable result in buccal MN assay and stated that it should be equal to 4,000.

Crucial for buccal MN assay is staining because all epithelial cells have different types of keratohyalins. Cell injury (cytotoxicity) which can take place due to smoking (because of cell exposure to cytotoxic/genotoxic substances in tobacco smoke) can increase production of these proteins in cells (which appear in cells as bodies which do not contain DNA). When DNA non-specific stains are used, they visualize these bodies which can mimic MN. This phenomenon was for the first time showed by Casartelli et al., (1997) and Casartelli et al., (2000). Further, this phenomenon was confirmed in our study with different staining techniques in the buccal cells of smokers (Nersesyan et al., 2006). In the buccal cell MN assay protocol and further guidelines, it is indicated that the presence of MN should be confirmed under fluorescent light because after this type of staining all bodies containing DNA fluorescent (Thomas et al., 2009; Bolognesi et al., 2013).

Hence, the study by Fahradi et al., (2017) contains triple limitation (incorrect stain, low number of scored cells and lack of MN confirmation under fluorescent light) and therefore, the results obtained in the study are not reliable. 

Another serious problem of this publication is enormous high level of MN. Indeed, it is indicated in Tables 1 and 2 that MN levels in non-smokers are 27.3‰ (2.73%) and 37.0%, 47.4% and 29.0% in smokers. These numbers are extremely, unusually high! Indeed, MN in epithelial buccal cells are very rare events. In 1999, Fenech et al., (1999) stated that the average MN frequencies in exfoliated buccal cells of healthy subjects are between 1.0 and 3.0‰. Later, it was reported by Bonassi et al., (2011) (based on database of 5,424 subjects) and Ceppi et al., (2010) (based on the data of 63 studies) that MN level in buccal cells of healthy unexposed persons are 0.74‰ (between 0.3 and 1.7%) and 1.10% (between 0.70 and 1.72%), respectively (Ceppi et al., 2010). In other words, in the study of Fahradi et al., (2017) the level of MN in non-exposed healthy subjects is much higher than in publications of other investigators. This level is between 24.9- and 36.9-fold higher compared with other data which is not possible, of course.

The authors stated in the abstract and also in the text of the article that “*differences were significant in smokers vs. nonsmokers for MN*” (in the original is written “*smopkers*”). But careful examination of the data presented in Table 2 shows that difference between smokers with history of ≤10 years and non-smokers (from Table 1) is not significant (29.0% and 27.3%, respectively). The authors wrote that “*statistical analysis was performed using the t-test*”. If so, the statistical significance of difference between mentioned data is t = [29.0 – 27.3 / √ (82 + 10.92)] = 1.7/13.5 = 0.12 which is far from critical value 1.96 to be significant at p < 0.05). It means that the difference between smokers with history of ≤10 years and non-smokers is not significant. In this case the authors should write that MN level increases significantly after 10 years of smoking, and duration smoking less than 10 years does not induce MN in buccal cells. The application of Student’s t test for statistical analysis of MN in buccal cells is not correct. Better to use non-parametric tests (Kruskal-Wallis and U-test Mann-Whitney). At least, the authors ought to normalize the data by means of log or square root transformation and then apply t-test. 

Fahradi et al., (2017) stated that “*karyorrhexis is a form of nuclear change in which nuclei are pyknotic or partially pyknotic*”. This is completely wrong statement because they mixed up two types of nuclear anomalies, i.e. karyorrhexis and pyknosis (Thomas et al., 2009; Bolognesi et al., 2013). 

In all legends to Figures 1 – 3 in which different types of nuclear anomalies are presented, is written that anomalies are “*Marked by an Arrow*”. But no one arrow is indicated. Also, the photos are of poor quality and it is not possible to see any anomaly in them. Instead of one cell there are several and it is absolutely not clear which cell the author mentioned. 

The authors stated that there are several reports concerning MN induction in smokers and mentioned following papers: Kamboj et al., (2007); Stich et al., (1982); Majer et al., (2001) and Rosin et al., (1987). All these papers are not relevant in this regard. Indeed, the paper by Majer et al. is comprehensive review, research papers by Kamboj et al., (2007) describes MN score in patients with squamous cell carcinoma and leukoplakia and Stich et al., (1982) concerns betel quid chewers. I could not find the paper by Rosin et al., (1987) but in the abstract presented in PubMed is written that the paper concerns tobacco and betel quid users in the Philippines and snuff users in the Northwest Territories. Instead they could cite the paper which they mentioned in another regard, namely Stich and Rosin (1983) concerning MN assay in heavy smokers. 

As for “*repair index*”, I am not sure about its usefulness. Indeed, this parameter is not indicated in the validated protocol. Instead of this index I suggest to the authors include into analysis scoring of basal cells. This parameter will show changes in the proliferation of buccal cells (Thomas et al., 2009). I hesitate if it should be expressed as %. Indeed, RI of non-smokers (Table 1) is 1.51 + 1.29/ 2.73 + 0.9 = 0.77. So it is mistake to state that RI is expressed in %.

In summary, Fahradi et al., (2017) carried out research work with 60 subjects but made some mistakes which are unfortunately quite common in case of ignoring the validated and standardized protocol for buccal MN cytome assay (Thomas et al., 2009). Consideration of all parameters suggested in the protocol will increase the reliability of the study and will give possibility to compare the results obtained in different laboratories.
